# Aflatoxin in Raw and Salt-Roasted Nuts (Pistachios, Peanuts and Walnuts) Sold in Markets of Tabriz, Iran

**DOI:** 10.5812/jjm.8674

**Published:** 2014-01-01

**Authors:** Alireza Ostadrahimi, Fereshteh Ashrafnejad, Abdolhassan Kazemi, Nafiseh Sargheini, Reza Mahdavi, Mohammadreza Farshchian, Sepideh Mahluji

**Affiliations:** 1 Nutritional Research Center, Faculty of nutrition, Tabriz University of Medical Sciences, Tabriz, IR Iran; 2 Liver and Gastrointestinal Diseases Center, Tabriz University of Medical Sciences, Tabriz, IR Iran; 3 Department of Biochemistry and Nutrition, Faculty of Nutrition, Tabriz University of Medical Sciences, Tabriz, IR Iran; 4 Department of Health of Environment, Tabriz University of Medical Sciences, Tabriz, IR Iran

**Keywords:** Aflatoxin, Nuts, Enzyme-Linked Immunosorbent Assay

## Abstract

**Background:**

Nuts are one of the main consumed snacks worldwide and also have an important role among Iranian's food habits. Natural contamination of nuts with aflatoxin is unavoidable and causes a special challenge for nuts safety and quality.

**Objectives:**

The purpose of this research was to study the aflatoxin contamination in commercially-available nuts (pistachio, walnut and peanut) in the markets of Tabriz, Iran.

**Materials and Methods:**

Sixty two samples of 50 g salt-roasted peanuts and pistachios and 109 samples of 50 g pure pistachios, walnuts and peanuts were collected from different areas of local markets. After the initial preparations, ELISA test was performed for Aflatoxin measurement.

**Results:**

Result showed that walnut (90%) and pure pistachio (2.3%) were the most and least contaminated samples, respectively. Mean aflatoxin contamination in the salt-roasted samples (19.88 ± 19.41 µg/kg) was significantly higher than the pure ones (6.51 ± 9.4 µg/kg) (P < 0.001). Respectively, 58.6%, 48.4% and 47.6% of salt-roasted pistachios, salt-roasted peanuts and walnut samples had aflatoxin contamination, which were more than the maximum tolerated level of Iran (MTL, 15 ppb).

**Conclusions:**

It was concluded that aflatoxin content of nuts should be monitored regularly to minimize the risk of aflatoxin hazard and ensure the food safety and quality.

## 1. Background

Fungi produce several metabolites and develop rapidly on different kinds of foods (1). These microorganisms can easily spread by wind, insects and raining, thus, survive in the environment (2). Molds naturally produce a wide range of metabolites, called mycotoxins. Mycotoxins can have toxic effects on humans and animals tissues and organs (3). They are mentioned as the 21st century concern due to their important pathogenic roles. The most toxic form of mycotoxins is called aflatoxin (AF). Studies have shown that AF contaminates some kinds of foods such as nuts, dried fruits, cereals and tea, since these groups are often exposed to the fungal infestations (4-6). According to the food and agriculture organization (FAO), globally, up to 25% of the foodstuff are contaminated with mycotoxins (7).

Among the nut products, pistachios are extremely contaminated with AF (8). In an Iranian study, the mean level of AF in pistachios was recorded as 7.3 ± 53.2 ng/g which was lower than the AF maximum tolerated level (MTL) of Iran (8). In another study in Saudi Arabia, the concentration of AF in peanuts was 28 μg/kg (9). The most important risk of AF for human and also animals is due to chronic dietary exposure (10). Various epidemiologic studies have indicated that AF, a serious concern to nuts safety and quality, causes human gastrointestinal disorders, hepatic neoplasm and liver cell carcinoma (3, 11). Even small amounts of AF in the foods, threaten the public health (1). However, countries try to control the levels of mycotoxins particularly AF in food and agricultural products; sometimes it is difficult to deal with several factors and lower the AF levels (12). Nuts AF is not influenced by temperature and remains active even in 160°C (13). In some countries like Iran, production and consumption of nuts (untreated and salt-roasted) have been increased (14, 15). Since nuts are known as healthy foods and also have pleasant tastes, people have a great tendency to consume them instead of other snacks such as chips and popcorns (16).

## 2. Objectives

Fungal contamination is currently a public health concern and there is a global trend to reduce its regarding problems. Considering the fact that nuts consumption is excessive in Iran (1), the aim of this study was to determine the AF levels of Iranian nuts such as untreated and salt-roasted pistachios and peanuts as well as untreated walnuts.

## 3. Materials and Methods

### 3.1. Samples

One hundred and nine samples of untreated nuts (pistachios, walnuts, and peanuts) and 62 samples of salt-roasted nuts (pistachios and peanuts) were collected randomly (50 g of each) (area sampling) from different parts of Tabriz city, delivered in sterile packets, moved to a laboratory and kept in a cool place (3 - 5ºC) maximum for three days. Nuts were surface-sterilized in 4% sodium hypochlorite for two minutes, diluted with distilled water three times to a concentration of 2%, rinsed in 100 mL distilled water and then let to dry.

### 3.2. Aflatoxin Analysis

Samples were analyzed by the ELISA method. EuroClone total AF ELISA test kits (EuroClone S.P.A: Italy, code: EEM002096 format 96 tests) were used for analysis. AF extraction was carried out according to the manufacturer's instructions (EuroClone S.P.A). For this purpose, 10 g of grinded samples was taken and 50 mL of 33% methanol solution (methanol: distilled water, 30:60) added, shaken for two minutes and then let settle for 15 minutes at room temperature. Then, the extract was filtered through a Whatman Nº 1 filter paper, the clear supernatant was diluted 1:2 with 33% methanol solution (1 mL + 1 mL) and samples were tested through ELISA kits instructions. The topical density was measured at 450 nm using ELISA - well plate reader. Evaluation of the ELISA data as well as the AF concentration were performed using the software program (EuroClone S.P.A: Italy).

### 3.3. Statistical Analysis

SPSS program version 11.5 was used for statistical analysis (SPSS Institute Inc. Chicago, Illinois). Descriptive statistical analysis was reported as Mean ± SD and percentages. Comparing the mean differences among the untreated nuts was done by analysis of variance (ANOVA). One sample T-test was used to compare the mean of AF in samples with MTL of Iran. Independent T-test was used to compare the AF means between the two groups of nuts. P value ≤ 0.05 was considered significant.

## 4. Results

The average recovery and standard deviation for AF levels were 102.13 ± 8.89 µg/kg. Mean of total AFs in untreated and salt-roasted samples were 6.51 ± 9.4 µg/kg and 19.88 ± 19.41 µg/kg, respectively. Table 1 presents the mean and range of total AF levels in the analyzed samples. Mean differences of the AF levels between salt-roasted and untreated nuts were significant (P < 0.001). Differences of the means between salt-roasted pistachios and peanuts were not significant (P = 0.42), but between pure pistachios and walnuts as well as walnuts and pure peanuts were significant (P < 0.001). Walnuts with 90.69% as well as roasted and pure with 2.3% contamination had the most and least AF incidences, respectively. Table 2 shows the percentages of contaminations with AF in the analyzed samples. 

**Table 1.** Mean and Range of the Total AF levels in the Analysed Samples

Types of Nuts	Mean ± SD, µg/kg	Range, µg/kg	Number of Analysed Samples
**Untreated ** ^**a**^	6.51 ± 9.4	0 - 38.1	109
Walnut	14.4 ± 8.4	0 - 38.1	43
Pistachio	0.48 ± 3.1	0 - 2.08	43
Peanut	3.03 ± 8.6	0 - 31.7	23
**Salt-Roasted ** ^**a**^	19.88 ± 19.41	0 - 52.3	62
Pistachio	22.02 ± 20.2	0 - 52.3	29
Peanut	17.99 ± 18.7	0 - 51.7	33

^a^ P < 0.001.

**Table 2.** Percentage of AF Incidence in Analyzed Samples

Types of Nuts	Number of Samples With AF ^a^ Contamination (%)	Number of Analyzed Samples
**Pure pistachio**	1 (2.3)	43
**Pure peanut**	4 (17.3)	23
**Pure walnut**	39 (90.69)	43
**Salt-roasted pistachio**	16 (55.17)	29
**Salt-roasted peanut**	5 (15.15)	33

^a^ Abbreviation: AF, aflatoxin.

**Figure 1.**
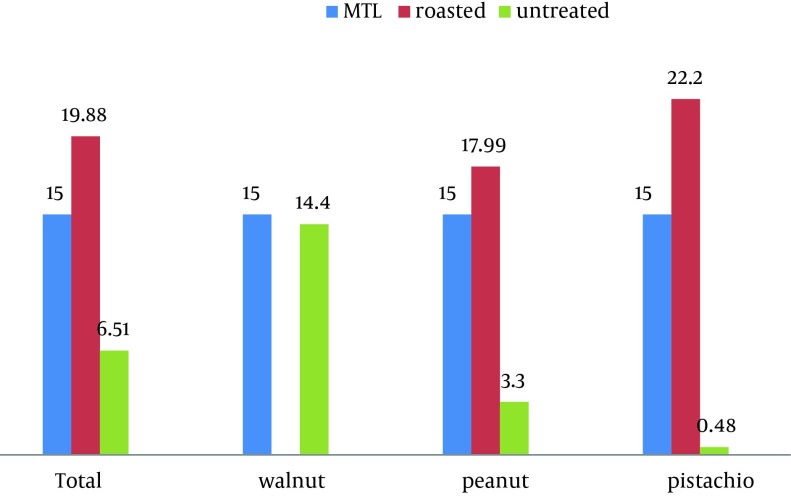
Comparison of Mean AF Levels (µg/kg) of Nuts With MTL of Iran (15 ppb)

Table 3 shows the numbers and percentages of samples containing AF more than 15 ppb (parts per billion). According to this Table, nuts containing AF of more than 15 ppb were considerable in the salt-roasted samples (pistachios: 58.62%, peanuts: 48.4%) and pure walnuts (47.6%). Figure 1 compares the mean of AF levels among nuts with MTL of Iran. Accordingly, mean of AF levels in the roasted pistachios was more than MTL (P < 0.05), but in untreated pistachios and peanuts, was significantly less than MTL (P < 0.001). In comparison to MTL of Iran, mean of total AF level in the untreated groups (6.51 ± 9.4 µg/kg) was significantly low (P < 0.001), while in the salt-roasted samples (19.88 ± 19.41 µg/kg) was high (P = 0.05). 

**Table 3.** Numbers and Percentages of Samples Containing AF > 15 ppb

Nuts	Number of Analyzed Samples	AF ^a^ containing samples, No. (%) ^b^
**Untreated walnuts**	43	20 (47.6)
**Untreated pistachios**	43	6 (2.3)
**Untreated peanuts**	23	1 (4.3)
**Roasted pistachios**	29	17 (58.62)
**Roasted peanuts**	33	16 (48.4)

^a^ Abbreviation: AF, aflatoxin.

^b^ number of samples with AF levels more than 15 ppb.

## 5. Discussion

Food contamination with AF is a serious health problem in the community (17). Nuts are subjects of this contamination among the food crops because of their composition and storage conditions (16, 18). The obtained results show that the mean of AF levels in the salt roasted group (19.88 ± 19.41 µg/kg) was more than untreated group (6.51 ± 9.4 µg/kg). In Malaysia, contamination range of nuts and nutty products with AF was 16.6 - 711 μg/kg (19). Research on Korean nuts showed that contamination with AF was in the range of 0.20-28.2 μg/kg (20), which was closer to the present study (0 - 38.1 μg/kg). In contrast to the findings of Nigerian scientists, our study showed that range of contamination with AF in roasted peanuts was 0 - 51.7 µg/kg, whereas in their study, it was 10 - 176 ppb in dry roasted ground nuts. This can be the result of predisposing conditions for the incidence of AF in Nigeria such as unfavorable storage conditions, bad distribution into the nylons, and cross infection by other products of the markets (15). 

A study in Zanjan (Iran) indicated that 60% of salted peanuts and 93.7% of pure samples were contaminated with AF (21), while findings of the present study showed that 15.15% of the roasted peanuts and 17.3% of pure samples were contaminated with AF. This can be the result of susceptible conditions such as high temperature, relatively high humidity, low light intensity, and long-term storage (22). Kladpan et al. (23) in Thailand stated that 3.6% of raw groundnuts and 50% of roasted groundnuts had AF contaminations of more than 20 ppb (MTL of AF levels set by the Thailand ministry of public health), their findings were closer to our study.

In Chun et al. (20) study, 10.6% of peanut samples contained AF, many of which were roasted. In the present study, the mean of AF contamination levels in walnuts was 14.4 ± 8.4 μg/kg, while in Qatar, walnut samples were free from AF (24). Juan in morocco reported that incidence of AF in walnuts was 30% (25), whereas in our study walnut contamination was 90.69%. According to the findings of researchers in Pakistan, shell-less walnuts had the maximum contamination level among all nut samples (11). It seems that high contamination of walnuts in the present study might be due to the fact that all collected samples were shell-less and some of them were damaged. Studies have shown that aflatoxin contamination in damaged and shell-less nuts is more than in-shell nuts (11, 26). 

Cheraghali et al. (8) reported that the total AF level was detected in 28.3% of pistachios with mean of 7.3 ± 53.2 µg/kg which was lower than MTL of Iran (15 ppb), while in the present study, incidence of AF was 2.3% in the pure pistachios and 55.17% in the salt-roasted ones and mean of AF levels was 0.48 ± 3.1 µg/kg in the pure and 22.02 ± 20.2 µg/kg in the salt-roasted pistachio samples. Result of an Algerian study showed that 64.5% of the analyzed pistachio samples were contaminated with AF at levels of 0.4 and 0.7 μg/kg (27). Higher levels of AF in the roasted samples of the present study might be due to the prolonged storage (28), moisture (29, 30), and suitable temperature for growth of mycotoxigenic molds (29). In addition, during the sample roasting procedure, fungi are probably destroyed, but their toxins (AF) are stable to dry heat, sometimes even up to 250°C. Hence, according to the findings of various studies, in Iran, long term storage of roasted nuts in bad conditions can cause AF contaminations in markets and stores.

Although HPLC is the best technique for AF measurement, in the present study, number of samples was high, and unfortunately our funding source was limited, thus ELISA was efficient. On the other hand, there were studies in which results of ELISA techniques were similar to HPLC (31, 32). Besides, ELISA is rapid, cost-effective, accurate, sensitive, easy to use, and convenient (32). High levels of AF in the food crops especially in nuts are probably due to the old and poor methods of manufacturing, storage, transition and marketing. It seems that long-term consumption of the AF-contaminated nuts has carcinogenic and toxigenic effects on the human health. Hence, in Iran, necessary steps should be taken by the health organization and other related agencies to minimize the AF contamination and also educate people about the danger of AF in nuts, which are favorable products and used as nourishing and safe snacks in the Iranian society.
